# Supplementary material for: From sequence to enzyme mechanism using multi-label machine learning
Source: BMC Bioinformatics. 2014 May 19;15:150. doi: 10.1186/1471-2105-15-150 (PMC4229970; doi:10.1186/1471-2105-15-150)
Supplement: Additional file 2 — Java code of ml2db. Additional file ml2db_code.tar.gz contains the Java source code to run the multi-label machine learning experiments and save the results to database. The code’s Javadoc is included. [file 1471-2105-15-150-S2.zip › additional file 2/ml2db/utils/doc/uk/ac/standrews/utils/main/stringutils/StringPool.html]

StringPool


JavaScript is disabled on your browser.


- Overview
- Package
- Class
- Use
- Tree
- Deprecated
- Index
- Help

- Prev Class
- Next Class

- Frames
- No Frames

- All Classes

- Summary:
- Nested |
- Field |
- Constr |
- Method

- Detail:
- Field |
- Constr |
- Method


uk.ac.standrews.utils.main.stringutils

## Class StringPool

- java.lang.Object
- - uk.ac.standrews.utils.main.stringutils.Pool
  - - uk.ac.standrews.utils.main.stringutils.StringPool

- All Implemented Interfaces:
  :   java.io.Serializable

  ---

    

  ```
  public class StringPool
  extends Pool
  ```

  Class

  Version:
  :   20 Jan 2012

  Author:
  :   Luna De Ferrari luna.deferrari@ed.ac.uk or
      luna.deferrari@st-andrews.ac.uk

  See Also:
  :   Serialized Form

- - ### Field Summary

    - ### Fields inherited from class uk.ac.standrews.utils.main.stringutils.Pool

      `m_debug`
  - ### Constructor Summary

    Constructors

    Constructor and Description

    `StringPool(int size)`
  - ### Method Summary

    Methods

    | Modifier and Type | Method and Description |
    | --- | --- |
    | `java.lang.String` | `getCanonicalVersion(java.lang.String str)` |

    - ### Methods inherited from class uk.ac.standrews.utils.main.stringutils.Pool

      `getSize`
    - ### Methods inherited from class java.lang.Object

      `equals, getClass, hashCode, notify, notifyAll, toString, wait, wait, wait`

- - ### Constructor Detail


    - #### StringPool

      ```
      public StringPool(int size)
      ```
  - ### Method Detail


    - #### getCanonicalVersion

      ```
      public java.lang.String getCanonicalVersion(java.lang.String str)
      ```


- Overview
- Package
- Class
- Use
- Tree
- Deprecated
- Index
- Help

- Prev Class
- Next Class

- Frames
- No Frames

- All Classes

- Summary:
- Nested |
- Field |
- Constr |
- Method

- Detail:
- Field |
- Constr |
- Method
